# Programming CRISPRi to control the lifecycle of bacteriophage T7

**DOI:** 10.3389/fmicb.2025.1497650

**Published:** 2025-02-12

**Authors:** Tobias Bergmiller

**Affiliations:** Department of Biosciences, Faculty of Health and Life Sciences, University of Exeter, Exeter, United Kingdom

**Keywords:** bacteriophage, CRISPRi, dCas9, fluorescent reporter, bacteriophage lifecycle

## Abstract

Clustered Regularly Interspaced Short Palindromic Repeats interference (CRISPRi), based on catalytically dead Cas9 nuclease of *Streptococcus pyogenes*, is a programmable and highly flexible tool to investigate gene function and essentiality in bacteria due to its ability to block transcription elongation at nearly any desired DNA target. In this study, I assess how CRISPRi can be programmed to control the life cycle and infectivity of *Escherichia coli* bacteriophage T7, a highly virulent and obligatory lytic phage. This is achieved by blocking the expression of critical host-dependent promoters and genes that are required for T7 genome translocation and lifecycle progression. Specifically, I focus on the promoters within the non-coding internalisation signal region and the *E. coli*-recognised promoter *C* controlling T7 RNA polymerase (T7 RNAP) expression. Fluorescent reporter assays reveal that CRISPRi targeting of promoters in the internalisation signal is only moderately effective, whereas the downregulation of the phage’s own T7 RNAP occurs very efficiently. Effects on the time to lysis were strongest when the left-most promoter on the leading end of the T7 genome or T7 RNAP was targeted. The stringency of the CRISPRi approach further improved when using multiplex sgRNAs to target multiple phage regions simultaneously, resulting in a 25% increase in the time to lysis and up to an 8-fold reduction in plaque size. Overall, this study expands dCas9-dependent CRISPRi as a flexible tool to non-invasively manipulate and probe the lifecycle and infectivity of otherwise native T7 phage.

## Introduction

Clustered Regularly Interspaced Short Palindromic Repeats interference (CRISPRi) is a powerful tool to study genotype–phenotype associations or bacteriophage (phage)-host interactions in bacteria (Qi et al., 2013; Peters et al., 2016; Peters et al., 2019; Mutalik et al., 2020; Rishi et al., 2020), and to engineer *de-novo* synthetic gene regulatory circuits and control (Kuo et al., 2020; Rueff et al., 2023). A commonly used CRISPRi approach originates from the CRISPR-Cas9 system of *Streptococcus pyogenes* and utilises a catalytically dead Cas9 (dCas9) nuclease that does not cleave DNA but sterically blocks transcription elongation by RNA polymerase, leading to repression or knockdown of the target gene (Qi et al., 2013). Transcription blockage requires a single guide RNA (sgRNA) that programmes dCas9 to bind to the non-coding strand at a specific DNA target containing an NGG protospacer-adjacent motif (PAM). Virtually any gene or non-coding region containing a PAM can thus be targeted by dCas9, and its expression level can be manipulated with very high specificity (Qi et al., 2013; Rousset et al., 2018; Rishi et al., 2020).

In bacteria, this approach requires only minimal engineering of the target strain by introducing an inducible dCas9 gene and sgRNA (Rousset et al., 2018; Rishi et al., 2020). The latter is commonly supplied on a low- to medium-copy plasmid. This enables straight-forward, cost-efficient, and flexible investigation of gene function across many bacterial species through the design and construction of sgRNAs or sgRNA libraries for high-throughput studies, thereby replacing the time-consuming construction of gene knockouts or transposon mutant libraries (Peters et al., 2019). Furthermore, the expression of more than one sgRNA enables multiplex approaches to manipulate several genetic targets simultaneously, to explore combinatorial effects (Ellis et al., 2023), and to engineer metabolic pathways and flux (Kim et al., 2017). Other engineering avenues have explored the use of dCas9 to activate transcription in bacteria by fusing transcriptional activation domains to dCas9 [termed CRISPRa-dCas9 (Dong et al., 2018)], or to construct more complex regulatory and repressilator-like circuits (Kuo et al., 2020; Rueff et al., 2023).

Until recently, dCas9-dependent CRISPRi has mainly been used to study and manipulate the genomic content or plasmids in bacteria (Rousset et al., 2018; Peters et al., 2019; Rishi et al., 2020), but not to target bacteriophage (phage) genomes to study phage gene function or to control their lifecycles. Phages are viruses that exclusively predate and kill bacteria with high specificity and efficacy, and they are of great interest for therapeutic applications to combat infections caused by multi-drug-resistant bacterial pathogens (Strathdee et al., 2023). Of great therapeutic interest are obligatory lytic phages, such as *Escherichia coli* phage T7, that lack the biphasic lysis-lysogeny lifecycle of temperate phages, and that eliminate their bacterial hosts with high specificity and efficacy. T7 is a highly virulent phage that has been extensively studied over the past decades (Calendar, 2006). Its host range can be synthetically expanded to other Gram-negative species (Yehl et al., 2019), and its small 40 kb genome can be reassembled using standard molecular techniques and rebooted *in-vivo* or *in-vitro* using cell-free systems (Levrier et al., 2024). This versatility makes T7 easy to manipulate and of great interest for therapeutic and biotechnological applications.

**Figure 1:**
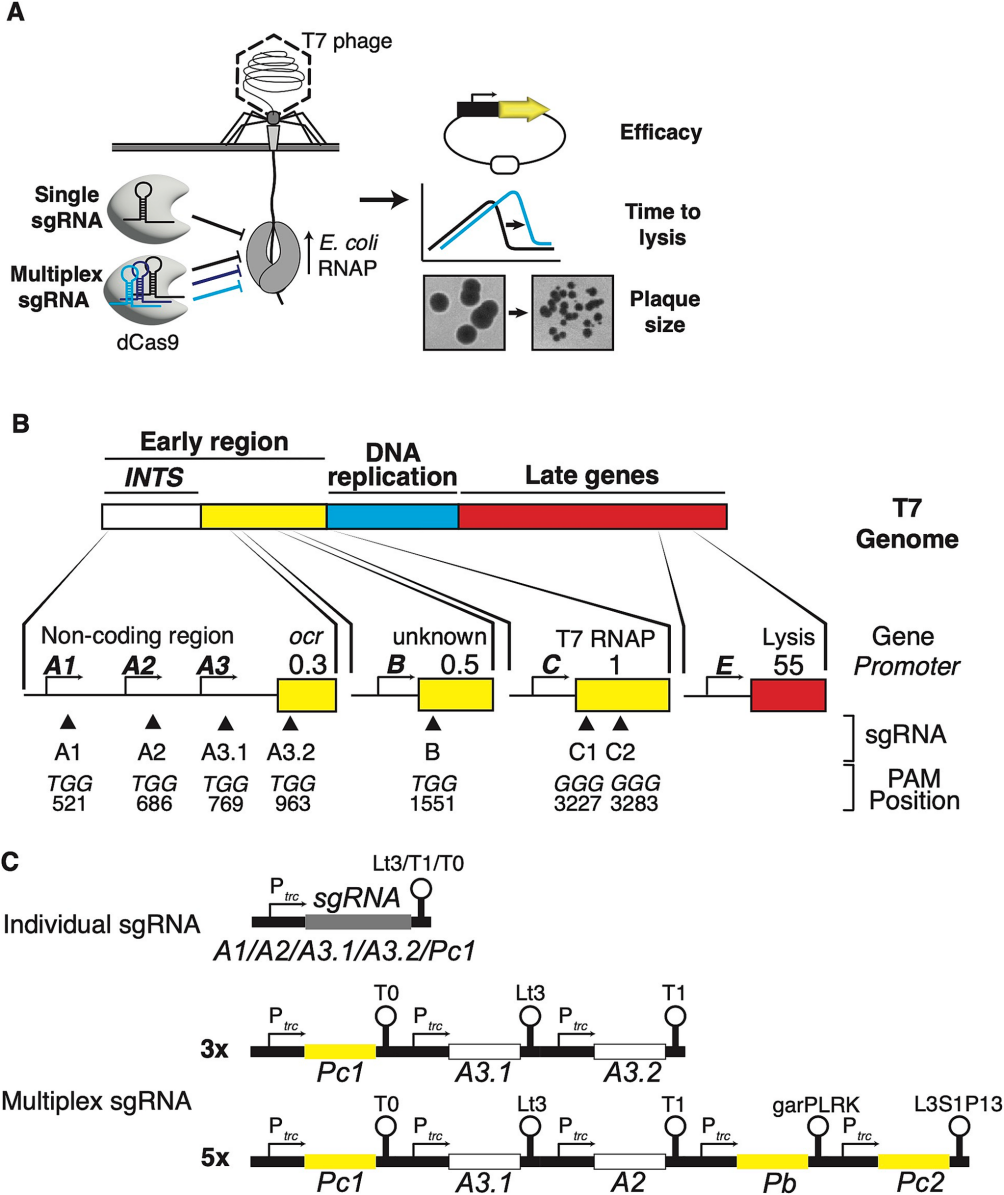
Targeting host RNAP-dependent phage promoters and genes to interfere with the lifecycle of T7 phage. **(A)** Schematic of DNA ejection by T7 phage and CRISPRi action. T7 ejects about 850 bp of its genome into the host cell which comprises the non-coding internalisation signal. *E. coli* RNAP binds to the strong promoters on the internalisation signal and catalyses further genome entry and transcription of early genes, leading to coupled internalisation and lifecycle execution. Using single or multiplex sgRNAs, dCas9 is programmed to interfere with *E. coli* RNAP at specific sites along the T7 phage genome. Fluorescent reporters are used to assay the efficacy of this approach using fluorescent knockdown experiments, and batch culture and plaquing experiments to quantify the impact of this approach on the time to lysis and infectivity of T7 phage. **(B)** Schematic of the T7 phage genome and its three modules (not to scale). The lower section shows the approximate positions of *E. coli* RNAP-dependent phage promoters and sgRNA binding (triangles), the nucleotide sequences of selected PAMs, and their exact positions on the T7 genome. The numbers refer to the position of the first nucleotides of the corresponding PAM. **(C)** Configuration of single sgRNAs and 3× and 5× multiplex sgRNAs. For 3× and 5× multiplex sgRNAs, different terminators are used for each individual promoter-sgRNA-terminator module (see main text and material and methods for details). sgRNA colour coding matches targets within *INTS* or T7 RNAP.

The T7 phage has a highly modular 40 kb genome, separated into three modules (Figure 1B). The first module is the early region, which includes the non-coding internalisation signal, genes required for hijacking the host, and the T7 RNA polymerase (RNAP). The second module is dominated by genes required for DNA replication, while the third module performs phage assembly and release functions. These modules are arranged in a chronological order of transcription events that occur during genome entry (Calendar, 2006). The T7 phage ejects approximately 850 bp of the left leading end of its genome, the internalisation signal, into the host cell by a virion-catalysed DNA ejection step (Pao and Speyer, 1973). Further translocation of the T7 genome strictly requires catalysis by the host’s RNAP, which may exert mechanical force during transcription (Zavriev and Shemyakin, 1982; Garcia and Molineux, 1995). This *E. coli* RNAP-dependent step is facilitated by an array of three strong phage promoters, *A1*, *A2*, and *A3*, that comprise the otherwise non-coding internalisation signal [or “early” region (Dunn and Studier, 1983)]. Once further genome entry has occurred, expression of the phage’s own T7 RNAP, initially transcribed by *E. coli* RNAP from promoter *C* on the T7 genome, leads to full genome internalisation and lifecycle execution (Garcia and Molineux, 1996). This culminates in host lysis and progeny release 11 min after DNA injection at 37°C.

Though the catalytically active CRISPR-Cas9 has been shown to cleave phage DNA efficiently (Lemay et al., 2017; Shen et al., 2018; Sun et al., 2024), relatively limited experimental evidence exists of the efficacy of *S. pyogenes* dCas9-based CRISPRi towards phages. Rousset et al. suggest that CRISPRi can target the genome of the temperate phage lamb (Rousset et al., 2018), while *in-vitro* studies of T7 RNAP show high efficacy of dCas9 in blocking transcription elongation of T7 RNAP (Widom et al., 2019). CRISPRi has also been shown to be effective in studying mycobacteriophage D29 (Barman et al., 2022). Recent reports demonstrated the use of dCas12a for functional studies of temperate phages Lambda and P1 in *E. coli* (Piya et al., 2023), and CRISPRi-ART based on dRfxCas13d for a range of different phages (Adler et al., 2023). However, the usability of the popular *S. pyogenes* dCas9-dependent CRISPRi system to manipulate the lifecycle or infectivity of T7 phage for functional studies or synthetic biology approaches such as engineering an infectivity switch (Chitboonthavisuk et al., 2022) remain largely unexplored. The synthetic biology approach requires genetic engineering of the phage, and the use of CRISPRi may unlock alternative paths of controlling phage infectivity or lifecycles.

In this study, I design, test, and quantify the efficacy of CRISPRi to interfere with or control the T7 phage lifecycle and infectivity by targeting important *E. coli* RNAP-dependent phage promoters and genes. I focus on the promoters in the early non-coding region comprising the internalisation signal and T7 RNAP, which is controlled by *E. coli* RNAP promoter *C*. I build fluorescent reporter plasmids and use the time to lysis and plaque size measurements to quantify the efficacy of CRISPRi and to interfere with the lifecycle of the T7 phage. This study is a proof-of-concept study that employs a limited set of rationally designed sgRNAs to highlight the use of the popular dCas9-dependent CRISPRi tool to tweak the infectivity of lytic phages without the requirement of phage engineering.

## Materials and methods

### Growth media and chemicals

Cloning and standard molecular biology techniques were performed using the strains outlined below, in LB broth (Melford, 10 g/L NaCl) and LB agar (prepared from LB broth and 1.5 g/L agar, Oxoid LP0011), with antibiotics added as indicated. Calcium chloride dihydrate (CaCl_2_, Sigma, C3306) and magnesium sulphate (MgSO_4_, Sigma, M2643) were prepared as 1 M stock solutions and sterilised by autoclaving. M9 salts (Formedium MMS0102) were prepared as 5× stock solution in Millipore water and autoclaved. To prepare M9 growth medium with glycerol, 5× M9 was added to sterile Millipore water to reach 1× concentration. Casamino acids (Formedium), MgSO_4_, and CaCl_2_ were added to final concentrations of 0.5×, 10 mM, and 0.1 mM, respectively, and sterile 50% glycerol stock was added to a final concentration of 0.2%. Chloramphenicol (Sigma C0378) and Ampicillin (Sigma A9518) were prepared as 15 mg/mL and 100 mg/mL stock solutions in 100% ethanol and Millipore water, respectively. The latter was filter-sterilised, and aliquoted stocks were stored at −20°C. L-arabinose (Sigma A3256) was prepared as a 1 M (15%) stock solution in Millipore water and sterile-filtered. Isopropyl beta-D-1-thiogalactopyranoside (IPTG, Sigma 16,758) was dissolved to 1 M concentration in Millipore water, sterile-filtered, and stored in small aliquots at −20°C. T7 phage stock was generously supplied by Dr. Remy Chait, University of Exeter, and was stored at 4°C in sterile-filtered SM buffer (200 mM NaCl_2_, 10 mM MgSO_4_, 50 mM Tris–HCl pH7.5).

### Strains, plasmids, and enzymes

Fluorescence knockdown experiments, phage lysis, and plaquing experiments were performed using TB549, a BW25113 derivative (F^−^ LAM^−^ *rrnB3* DE*lacZ*4787 *hsdR*514 DE(*araBAD*)567 DE(*rhaBAD*)568 *rph-1*) with a single-copy insertion of P_araBAD_-dCas9 into *att*HK022. Promoter strength comparisons in the absence of dCas9 and sgRNAs were performed in BW25113. Single-copy YFP fluorescence knockdown was conducted in TB566, which carries PR-YFP in *att*P21, using pZS*1-P_araBAD_-dCas9 as the source for dCa9 expression. Strains were grown at 37°C for standard molecular methodologies and for fluorescence knockdown experiments. T7 time-to-lysis experiments were conducted at 30°C to increase the contrast of time-to-lysis between induced and non-induced cultures, and plaque size and efficiency of plating (EOP) experiments were conducted at 22°C to prevent fusion of fast-growing T7 plaques. Strains were cryopreserved at −80°C in 15% sterile glycerol, and cultures were grown from freshly streaked individual colonies on agar plates.

Plasmid construction was conducted using the isothermal Gibson Assembly method and New England Biolabs (NEB) Gibson Assembly Master Mix (#E2611). Assemblies were transformed into commercially available and chemically competent cells, NEB5α, following the manufacturer’s guidelines. pAH68-frt-cat-P_araBAD_-dCas9, an R6K ori plasmid (Haldimann and Wanner, 2001; Bergmiller et al., 2017) was constructed using the pir + strain DH5αpir. Inserts of all assembled plasmids were verified using Sanger sequencing or whole plasmid sequencing with Oxford Nanopore technology (ONT, Eurofins, Germany). pZA3 (ChlorR), pZS12-YFP Venus (AmpR), and pZS12* plasmids with p15a, SC101, and SC101* origins of replication (Lutz and Bujard, 1997) were a gift from Calin Guet (Institute of Science and Technology, Klosterneuburg, Austria).

### Strain and plasmid construction

To construct pAH68frtcat-P_araBAD_-dCas9 and TB549, P_araBAD_-dCas9 was amplified from pZA3-P_araBAD_-dCas9 (Bergmiller lab strain collection) with primer pair dCas9 FW and dCas9 RW and assembled with a PCR product of pAH68-frt-cat that was amplified with primers pAH68frt-cat FW and pAH68frt-cat RW. dCas9 was originally amplified from plasmid pJMP1159 (supplied by Addgene number #119250) removing the myc tag (Peters et al., 2019). The sequence of P_araBAD_ was taken from pLA2 (Haldimann and Wanner, 2001), which has its own native ribosome binding site. The sequence of the resulting plasmid pAH68-frt-cat-P_araBAD_-dCas9 was verified by whole-plasmid sequencing using Oxford Nanopore Technologies (ONT) (Eurofins, Germany). pAH68frtcat-P_araBAD_-dCas9 was then inserted into *att*HK022 of BW25113 using pAH69 and its single-copy status was verified by polymerase chain reaction (PCR) following previously described methods (Haldimann and Wanner, 2001). The Chloramphenicol resistance marker was subsequently cured using the helper plasmid pCP20 (Cherepanov and Wackernagel, 1995), resulting in the AmpS ChlorS strain TB549.

pZS*1-P_araBAD_-dCas9 was constructed using PCR amplification of P_araBAD_-dCas9 from pAH68frtcat-P_araBAD_-dCas9 and Gibson-assembled with a PCR product of pZS*12 (a gift of Calin Guet, IST Austria, Austria).

TB566 carries the genotype of BW27784 (Khlebnikov et al., 2001), which was P1-transduced with a constitutively expressed YFP (Cox et al., 2010) driven by the strong lambda PR promoter inserted into *att*P21 (Bergmiller et al., 2017) and cured of chloramphenicol resistance using pCP20 (*att*P21::PR-YFP::FRT).

Fluorescence reporter plasmids were constructed by generating a PCR product of pZS12-YFP Venus removing P_LlacO1_ using primers pZS1 FW and pZS1 RW. T7 phage fragments were synthesised as gBlocks by Integrated DNA Technologies (IDT, Belgium) with overlapping matching ends and Gibson-assembled with the pZS12-YFP Venus PCR product. All cloned T7 DNA fragments are listed in Table 1. Briefly, the *INTS* fragment spanned promoters *A1*, *A2*, and *A3*, and a short fragment of *ocr* with an in-frame stop codon. All other T7 DNA fragments included upstream and downstream sequences, and all fragments of coding regions were designed to have an in-frame stop codon. These fragments were then placed in front of the ribosome binding site of YFP Venus using Gibson assembly, and all inserts were verified by Sanger sequencing using primer pZS1-AmpR-seq.

**Table 1:** T7 *E. coli* RNAP promoter fragments.

Internalisation signal fragment (*INTS*)
TATCACGAGGCCCTTTCGTCTTCACCTCGAGTTTAAAATTTATCAAAAAGAGTATTGACTTAAAGTCTAACCTATAGGATACTTACAGCCATCGAGAGGGACACGGCGAATAGCCATCCCAATCGACACCGGGGTCAACCGGATAAGTAGACAGCCTGATAAGTCGCACGAAAAACAGGTATTGACAACATGAAGTAACATGCAGTAAGATACAAATCGCTAGGTAACACTAGCAGCGTCAACCGGGCGCACAGTGCCTTCTAGGTGACTTAAGCGCACCACGGCACATAAGGTGAAACAAAACGGTTGACAACATGAAGTAAACACGGTACGATGTACCACATGAAACGACAGTGAGTCACCACACTGAAAGGTGATGCGGTCTAACGAAACCTGACCTAAGACGCTCTTTAACAATCTGGTAAATAGCTCTTGAGTGCATGACTAGCGGATAACTCAAGGGTATCGCAAGGTGCCCTTTATGATATTCACTAATAACTGCACGAGGTAACACAAGATGGCTATGTCTAACATGACTTACAACAACGTTTTCGACCACGCTTACGAAATGCTGAAAGAAAACATCCGTTATGATGACATCCGT**TAA**GAATTCATTAAAGAGGAGAAAGGTA
Promoter *B*
GAGGCCCTTTCGTCTTCACCTCGAGAAAACAACAAGGCAATAGCTTTAGAATCTGCTGAGTGATAGACTCAAGGTCGCTCCTAGCGAGTGGCCTTTATGATTATCACTTTACTTATGAGGGAGTAATGTATATGCTTACTATCGGTCTACTCACCGCTCTAGGTCTAGCTGTAGGTGCATCCTTTGGGAAGGCTTTAGGTGTAGCTGTAGGTTCCTACTTT**TAA**AGAATTCATTAAAGAGGAGAAAGGTA
Promoter *C*
GAGGCCCTTTCGTCTTCACCTCGAGAACTTGACGCAATGTTAATGGGCTGATAGTCTTATCTTACAGGTCATCTGCGGGTGGCCTGAATAGGTACGATTTACTAACTGGAAGAGGCACTAAATGAACACGATTAACATCGCTAAGAACGACTTCTCTGACATCGAACTGGCTGCTATCCCGTTCAACACTCTGGCTGACCATTACGGTGAGCGTTTAGCTCGCGAACAGTTGGCCCTTGAGCATGAGTCTTACGAGATGGGTTAA**TAA**GAATTCATTAAAGAGGAGAAAGGTA
Promoter *E*
GAGGCCCTTTCGTCTTCACCTCGAGCGGTCTTACGGATGATGATATTTACACATTACAGTGATATACTCAAGGCCACTACAGATAGTGGTCTTTATGGATGTCATTGTCTATACGAGATGCTCCTACGTGAAATCTGAAAGTTAACGGGAGGCATTATGCTAGAATTTTTACGTAAGCTAATCCCTTGGGTTCTCGCTGGGATGCTATTCGGGTTAGGATGGCAT**TAA**GAATTCATTAAAGAGGAGAAAGGTA

Underlined sequences are homologous sequences to the pZA3 PCR product for Gibson assembly. In bold are stop codons to terminate the translation of orf fragments.

All sgRNAs were designed following the method described in Banta et al. (2020) and cloned into pZA3. Briefly, sgRNA sequences were selected either by identifying PAMs within the non-coding *INTS* region and designing sgRNA such that they would target the non-template strand 20–60 bps downstream of annotated −10/35 regions, or by using a Python script (Banta et al., 2020) for the coding regions of gp1 (T7 RNAP) and gp0.5. All sgRNA sequences are listed in Table 2. The constructs were then designed such that sgRNAs were expressed by a constitutive version of P_trc_ lacking lacO sites (Banta et al., 2020) followed by a “handle” sequence and a strong transcriptional terminator. Furthermore, sgRNAs A3.1, A3.2, and C were designed as gBlocks with overlapping matching ends to the pZA3 PCR product generated with primers pZA3 FW and pZA3 RW and terminators Lt3, T1, and T0. sgRNAs A1, A2, YFPI, YFPII, and the Control sgRNA (a random 20 bp DNA sequence with 50% GC content) were then constructed using the promoter C sgRNA plasmid pZA3-C as PCR template (amplified with pZA3-C FW and pZA3-C RW) and by assembling pZA3-A1, pZA3-A2, pZA3-YFPI, pZA3-YFPII, and pZA3-Control by using single-stranded oligonucleotides that had matching ends to the pZA3-C PCR product.

**Table 2:** sgRNA sequences.

Control	GTAGCGAAAGATCAAGGCGA
A1	GACCCCGGTGTCGATTGGGA
A2	TTGTTTCACCTTATGTGCCG
A3.1	GACCGCATCACCTTTCAGTG
A3.2	CTTTCAGCATTTCGTAAGCG
C1	GGTCAGCCAGAGTGTTGAAC
C2	CTCGTAAGACTCATGCTCAA
B	TACACCTAAAGCCTTCCCAA
YFP I	CACCAGACACGGAAAATTTG
YFP II	CGGAACAGGCAGTTTGCCGG
3× multiplex sgRNA construct	
GGATCCTAGGCGTATCACGAGGCCCTTTCGTCTTCATTGACAATTAATCATCCGGCTCGTATAATGTGTGGGGTCAGCCAGAGTGTTGAACGTTTAAGAGCTATGCTGGAAACAGCATAGCAAGTTTAAATAAGGCTAGTCCGTTATCAACTTGAAAAAGTGGCACCGAGTCGGTGCTTTTTTTTGTACGGGGATTAGCCAAGACGTCTAATGATCACCTCAGAACTCCATCTGGATTTGTTCAGAACGCTCGGTTGCCGCCGGGCGTTTAGCCTGCCTGTGTTTACACTGTCCTGCGGCTACCCATGGCCTGAAATCCAGCTCGTGTCAAGCCATTGCCTCTCCGGGACGCCGCTTGACAATTAATCATCCGGCTCGTATAATGTGTGGGACCGCATCACCTTTCAGTGGTTTAAGAGCTATGCTGGAAACAGCATAGCAAGTTTAAATAAGGCTAGTCCGTTATCAACTTGAAAAAGTGGCACCGAGTCGGTGCTTTTTTTTTCGGTAACGTACGCTCTAGCCACAAAGCGAGGCTGGGTATTTCCCGGCCTTTCTGTTATCCGAAATCCACTGAAATTTCCGCGAATGACAACAGACAGATCCCAATGTCGCTTTCGTCGTCAGCAACTCCGTTTTCACTATGCACGCTGTTTGACAATTAATCATCCGGCTCGTATAATGTGTGGCTTTCAGCATTTCGTAAGCGGTTTAAGAGCTATGCTGGAAACAGCATAGCAAGTTTAAATAAGGCTAGTCCGTTATCAACTTGAAAAAGTGGCACCGAGTCGGTGCTTTTTTTTTGCGCGCTGATGCGAATCCTGGCATCAAATAAAACGAAAGGCTCAGTCGAAAGACTGGGCCTTTCGTTTTATCTGTTGTTTGGCCAGTGCCAAGCTTGCATGC	
5× multiplex sgRNA construct	
GGATCCTAGGCGTATCACGAGGCCCTTTCGTCTTCATTGACAATTAATCATCCGGCTCGTATAATGTGTGGGGTCAGCCAGAGTGTTGAACGTTTAAGAGCTATGCTGGAAACAGCATAGCAAGTTTAAATAAGGCTAGTCCGTTATCAACTTGAAAAAGTGGCACCGAGTCGGTGCTTTTTTTTGTACGGGGATTAGCCAAGACGTCTAATGATCACCTCAGAACTCCATCTGGATTTGTTCAGAACGCTCGGTTGCCGCCGGGCGTTTAGCCTGCCTGTGTTTACACTGTCCTGCGGCTACCCATGGCCTGAAATCCAGCTCGTGTCAAGCCATTGCCTCTCCGGGACGCCGCTTGACAATTAATCATCCGGCTCGTATAATGTGTGGGACCGCATCACCTTTCAGTGGTTTAAGAGCTATGCTGGAAACAGCATAGCAAGTTTAAATAAGGCTAGTCCGTTATCAACTTGAAAAAGTGGCACCGAGTCGGTGCTTTTTTTTTCGGTAACGTACGCTCTAGCCACAAAGCGAGGCTGGGTATTTCCCGGCCTTTCTGTTATCCGAAATCCACTGAAATTTCCGCGAATGACAACAGACAGATCCCAATGTCGCTTTCGTCGTCAGCAACTCCGTTTTCACTATGCACGCTGTTTGACAATTAATCATCCGGCTCGTATAATGTGTGGTTGTTTCACCTTATGTGCCGGTTTAAGAGCTATGCTGGAAACAGCATAGCAAGTTTAAATAAGGCTAGTCCGTTATCAACTTGAAAAAGTGGCACCGAGTCGGTGCTTTTTTTTTGCGCGCTGATGCGAATCCTGGCATCAAATAAAACGAAAGGCTCAGTCGAAAGACTGGGCCTTTCGTTTTATCTGTTGTTCAACAGCTGTCTAGCAGTTCTAATCTTTTGCCATCGTCGTAAAAGCCTCCAAGAGATTGATCATACCTATCGGCATTGACAATTAATCATCCGGCTCGTATAATGTGTGGTACACCTAAAGCCTTCCCAAGTTTAAGAGCTATGCTGGAAACAGCATAGCAAGTTTAAATAAGGCTAGTCCGTTATCAACTTGAAAAAGTGGCACCGAGTCGGTGCTTTTTTTTTATCTAACTATCCCCTATGTCGGTCAGTTTCACCTGTTTTACGTAAAAACCCGCTTCGGCGGGTTTTTACTTTTGGCGCAAGGGTCGTGAAGTCGGTTCCTTCAATGGTTAAAAATCAAAGGCTCACTGTGCAGACTGGAGCGCCCATCTATTGACAATTAATCATCCGGCTCGTATAATGTGTGGCTCGTAAGACTCATGCTCAAGTTTAAGAGCTATGCTGGAAACAGCATAGCAAGTTTAAATAAGGCTAGTCCGTTATCAACTTGAAAAAGTGGCACCGAGTCGGTGCTTTTTTTTTGGCTTGGGTCGAGATAAAATTTTTGTTATCAATAAAAAAGGCCCCCCGTTAGGGAGGCCTTATTGTTCGTCTGGCCAGTGCCAAGCTTGCATGCCTGGGATCC	

3× and 5× multiplex sgRNA constructs were synthesised as full-size constructs by GenScript (GenScript Biotech, UK), cut out from the delivered plasmid using BamHI, and Gibson-assembled into pZA3. Multiplex constructs were designed such that each P_trc_-sgRNA-terminator module was preceded by a 75 bp random DNA sequence and either 3 or 5 different terminator sequences were chosen to minimise sequence redundancy. For 3×, terminators were T0-Lt3-T1, and for 5× T0-Lt3-T1-garPLRK-L3S1P13. pZA3 plasmids were all verified by Sanger sequencing using primer pZA3-seq.

All plasmid DNA sequences are available as GenBank files in the Supplementary material. DNA sequences of oligonucleotides can be found in Table 3, all plasmids and strains are listed in Table 4.

**Table 3:** Oligonucleotides.

dCas9 FW	CGTGGCCAGTGCCAAGCTTGCATGCCTGCAGCCGCCATTCAGA
dCas9 RW	ATCAGTGATAAGCTGTCAAACATGATTAGTCACCTCCTAGCTGAC
pAH68frt-cat FW	TCATGTTTGACAGCTTATCA
pAH68frt-cat RW	GCATGCAAGCTTGGCACTGG
pZA3 FW	CTGCAGCCGCCATTCAGA
pZA3 RW	TGAAGACGAAAGGGCCTCG
pZS1 FW	GAATTCATTAAAGAGGAGAA
pZS1 RW	CTCGAGGTGAAGACGAAAGG
pZS1-AmpR-seq	CGACACGGAAATGTTGAATA
pZA3-seq	AGCGAGGAAGCGGAATATAT
pZA3-C RW	CCACACATTATACGAGCC
pZA3-C FW	GTTTAAGAGCTATGCTGGAA
dCas9-pZS FW	CCGCCATTCAGAGAAGAA
dCas9-pZS RW	TTAGTCACCTCCTAGCTGA
pZS1-dCas9 FW	TTGAGTCAGCTAGGAGGTGACTAAAAGCTTAATTAGCTGAGTCTAG
pZS1-dCas9 RW	ATTGGTTTCTTCTCTGAATGGCGGGTGAAGACGAAAGGGCCT

**Table 4:** Plasmids and strains.

BW25113	F^−^ LAM^−^ *rrnB3* DE*lacZ*4787 *hsdR*514 DE(*araBAD*)567 DE(*rhaBAD*)568 *rph-1.* Bergmiller Lab strain collection
TB549	*att*HK022::P_araBAD_-dCas9::FRT F^−^ LAM^−^ *rrnB3* DE*lacZ*4787 *hsdR*514 DE(*araBAD*)567 DE(*rhaBAD*)568 *rph-1*
TB566	*att*P21::PR-YFP::FRT F^−^ LAM^−^ *rrnB3* DE*lacZ*4787 *hsdR*514 DE(*araBAD*)567 DE(*rhaBAD*)568 *rph-1* DE(*araH-araF*)570(::FRT) ∆*araEp*-532::FRT *phiP_cp18_araE*533 (Khlebnikov et al., 2001); Bergmiller Lab strain collection
pZA32 (ChlorR)	Calin Guet lab collection
pZS12-YFP Venus (AmpR)	Calin Guet lab collection
pZS*12	Calin Guet lab collection
pCP20	(Cherepanov and Wackernagel, 1995) Bergmiller lab strain collection
pAH69	(Haldimann and Wanner, 2001) Bergmiller Lab strain collection
pAH68frt-cat (ChlorR)	(Bergmiller et al., 2017) Bergmiller lab strain collection
pZA3-Para-dCas9	Bergmiller lab strain collection
pAH68frt-cat-P_araBAD_-dCas9	The current study
pJMP1159	dCas9 template (Peters et al., 2019), from Addgene
pZA3-Control	The current study
pZA3-A1	This study
pZA3-A2	This study
pZA3-A3.1	This study
pZA3-A3.2	This study
pZA3-C	This study
pZA3-3×	This study
pZA3-5×	This study

### Growth rate experiments

To estimate their doubling time, biological duplicates of bacterial strains were grown overnight to saturation, and when required, antibiotics were added to select for plasmids. Overnight cultures were then split into three technical replicates and diluted 1:200 into fresh broth in transparent 96well microtiter plates (Greiner #655161), again with fresh antibiotics added to select for plasmids, and plates were sealed using clear adhesive film (Fisher Scientific #15963620). The plates were then incubated at 37°C and optical density at 600 nm was measured every 5 min in a BMG Clariostar Plus. Before each measurement, the plate was shaken double-orbitally at 600 rpm for 10 s. Growth rates and doubling times were then estimated using a custom-made R script that fits a linear slope to the steepest part of log-transformed data over five time points using an *R*^2^ value of 0.98. Two separate doubling times for the initial *INTS* fluorescent reporter plasmid were estimated by fitting curves over each of five time points to the two separate growth phases as shown in Supplementary Figure 2. Growth rates of individual promoter constructs in the absence of sgRNA plasmids were measured in biological quadruplicates, while TB549 with or without sgRNA plasmids and dCas9 induction was assessed in biological duplicates with technical triplicates (Tables 3, 4).

### Fluorescent reporter strength and knockdown experiments

Fluorescent knockdown experiments using reporter plasmids were conducted in TB549 cells. To do so, TB549 was sequentially transformed with matching pZA3-sgRNA plasmids or the pZA3-Control plasmid and pZS1 reporter plasmids. Overnight cultures of double transformants were inoculated 1:200 into fresh LB broth with antibiotics in black 96well microtiter plates with clear bottom (Greiner #655090), sealed with clear adhesive film, and placed in a temperature-controlled BMG Clariostar Plus plate reader at 37°C. OD_600nm_ and YFP fluorescence readings (515 nm excitation, 530 nm emission) using the enhanced sensitivity range were taken every 5 min, preceded by 10 s of shaking in a double-orbital pattern at 600 rpm. After subtracting the blank values of control wells, fluorescence data was normalised by OD_600nm_ (Fluorescence/OD_600nm_).

Promoter strength was measured for each of the four independent biological replicates, and uninduced pZS12-YFP Venus, used to normalise promoter strength data, was measured in biological triplicates. To compare promoter strength across fluorescent reporters in the absence of sgRNA plasmids, single time points at 100 min were analysed and normalised to the mean fluorescence of TB549 harbouring uninduced pZS12-YFP Venus.

Fluorescent knockdown experiments were performed in biological duplicates that were split into two sets of technical triplicates into which either no L-arabinose (controls, no dCas9 expression) or 0.1% L-arabinose was added (dCas9 expression and fluorescence knockdown), yielding a total of six control cultures and six fluorescence knockdown cultures for each fluorescent reporter-sgRNA combination. To analyse the knockdown efficiency of fluorescent reporters, data was normalised to the mean of respective controls lacking arabinose. Data from timepoint 400 min (early stationary phase) were analysed for *INTS* and promoter *C* knockdown. For chromosomally inserted PR-YFP knockdown, data from 350 min (knockdown in M9 Glycerol) and 250 min (knockdown in LB) were analysed. Arabinose-dependent knockdown (0.01, 0.05, and 0.1% arabinose) of promoter *C* was measured in biological triplicates.

The choice of time point for quantifying promoter strength or knockdown efficiency affects the values that are employed to quantify these parameters. Overall, the time points were chosen such that fluorescence levels had stabilised or plateaued, which occurs during the early stationary phase. This is likely driven by the dilution of highly expressed and otherwise proteolytically stable YFP upon dCas9 knockdown. For some of the data, especially for the *INTS*-YFP construct, variation arises in fluorescent levels into the later stationary phase which can be caused by cell death or other unknown factors. Promoter strength experiments are less affected by equilibrating YFP levels as no knockdown occurs, and thus a robust time point to assess fluorescent levels is the mid-to-late exponential phase assuming near steady-state growth and an equilibrium between YFP production and dilution at this growth phase.

To compare fluorescent reporters and knockdown efficiencies on plates which serve as visual guides to compare knockdown efficiencies, agar plates with antibiotics and with or without 0.1% L-arabinose were sectored, and individual colonies replica-streaked onto separate sectors. Images of plates were taken using a custom-made fluorescent macroscopic imager [“macroscope” (Chait et al., 2010)], and their brightness and contrast were adjusted on a linear scale using ImageJ.

### T7 phage time to lysis experiments and data analysis

All T7 phage lysis experiments to determine the time to lysis were performed in transparent 96-well microtiter plates (Greiner #655161) in a BMG Clariostar Plus plate reader at 30°C. Biological duplicates of overnight cultures were diluted 1:100 into fresh LB with 0.1 mM CaCl_2_ and 10 mM MgSO_4_ and grown with or without 0.1% L-arabinose for 2.5 h reaching OD_600nm_ of 1. Then, a T7 phage dilution series was made in a 96-well plate containing columns of LB with 0.1 mM CaCl_2_ and 10 mM MgSO_4_ and with or without 0.1% arabinose, cultures split into technical triplicates, and cells added such that they were diluted 1:10 into wells with three different phage dilutions (10^−8^, 10^−9^ and 10^−10^ PFU). OD_600nm_ measurements were taken every 5 min and preceded by a 10 s 600 rpm double-orbital shake for 300 min. To find the point of culture lysis, the inflection points at which the slope turns negative were determined by fitting a slope using a sliding window of four time points to OD_600nm_ growth curves. The interception point of slopes with the X-axis was then determined using the Excel function “Intercept.” The onset of culture lysis was found to be increasingly variable at higher dilutions of 10^−9^ and 10^−10^ PFU due to extremely low phage numbers and multiplicities of infection (MOI), and subsequently, data from wells with 10^−8^ PFU were analysed (equalling an MOI of 10^−5^). The change in time to lysis was then calculated by normalising the time to lysis of cultures grown with 0.1% L-arabinose to the mean time to lysis of control cultures lacking L-arabinose.

### Top agar experiments

Efficiency of plaquing (EOP) and plaque size experiments were performed using the spot dilution method (Adams, 1959). Briefly, top agar (0.5% LB agar) was stored in 5 mL aliquots in a 50°C water bath with 0.1 mM CaCl_2_, 10 mM MgSO_4_, 15 μg/mL Chloramphenicol and with or without 0.1% L-arabinose. Strains carrying sgRNA plasmids were cultured with or without 0.1% arabinose for 3.5 h. Then, 1 mL of bacterial culture was added to the top agar, the tube inverted, and its content was poured on top of LB agar plates and allowed to dry. Thereafter, 3 μL of phage dilution was spotted onto the top agar, allowed to dry, and plates incubated at 22°C for 20 h. Images of plates were taken using a BioRad GelDock imager, and plaque area was measured using the oval selection tool in ImageJ. Three independent biological replicates were performed, and at least three plaques of each plate were measured.

## Results

### sgRNA selection and construction

Following T7 phage genome annotations and prior knowledge of the T7 phage lifecycle (Chan et al., 2005), I selected the reportedly strongest and most critical *E. coli* RNAP-dependent phage promoters *A1*, *A2*, and *A3*, comprising the non-coding internalisation signal (*INTS* hereafter), and the T7 RNAP gene *gp1* which is controlled by *E. coli* RNAP-dependent promoter *C*, as main targets for CRISPRi (Figures 1A,B). The rationale was that blocking these promoters and gene products interferes with the T7 phage lifecycle at different stages, allowing to control or interfere with its progression. More specifically, the hypothesis was that sterically blocking *INTS* may slow down or even abolish T7 infection by hindering the *E. coli* RNAP transcription-dependent T7 genome translocation process. Furthermore, the expression of early genes that are required to overcome host defence systems and to shut off host transcription is driven by the *INTS* promoters (Dunn and Studier, 1983). Blocking expression of T7 RNAP was another prime target of this approach, which followed the idea that blocking transcription of T7 RNAP may hinder the progression of the T7 lifecycle to later stages which are entirely T7 RNAP-dependent.

I consequently designed a set of sgRNAs using published design principles and where possible, an automated workflow (Banta et al., 2020). The sgRNAs were chosen such that dCas9 homes in on the 5′-end of the coding region of *gp1* which is controlled by promoter *C* (sgRNA C), and on *INTS* at several positions (Figure 1C). All sgRNAs were 20 nt long and targeted the non-template strand, which has been shown to effectively cause a strong roadblock for RNAP [(Qi et al., 2013; Cui et al., 2018; Rishi et al., 2020) see Material and Methods for details]. To minimise variations in efficacy arising from differences in design, sgRNAs towards *INTS* were designed to bind 20-60 bp downstream of the annotated transcription start sites of *A1*, *A2*, and *A3* (sgRNAs A1, A2, and 3.1), and a fourth sgRNA (sgRNA 3.2) targeting *gp0.3* (*ocr*) within the 5′ end of the open reading frame. Of note, the sgRNA towards *C* was designed to bind within the *gp1* coding region instead of directly targeting promoter *C* to have a maximal knockdown effect on T7 RNAP expression (Banta et al., 2020). The sgRNAs were then cloned into a medium copy sgRNA expression plasmid and expressed constitutively (see Material and Methods section).

I also constructed two multiplex sgRNA arrays consisting of 3 (3×) and 5 (5×) sgRNAs to target *INTS* and *gp1* at several sites simultaneously (Figure 1C). To maximise the effect of the 5× multiplex sgRNA approach, the *E. coli* RNAP promoter *B* controlling *gp0.5* was included as a multiplex target. Promoter *B* can have an accessory function for T7 genome internalisation (Garcia and Molineux, 1996), and it controls the expression of *gp0.5* whose function remains poorly understood. In addition, I constructed strain TB549 that harbours a chromosomally inserted single-copy and arabinose-inducible P_araBAD_-dCas9 construct (Haldimann and Wanner, 2001). Expression of dCas9 in TB549 by adding arabinose had no measurable effects on growth rate. Similarly, constitutive expression of sgRNAs in TB549, both in the absence and presence of dCas9 expression showed no negative effects on growth (Supplementary Figure 1).

### Quantifying CRISPRi efficacy using fluorescent reporters

**Figure 2:**
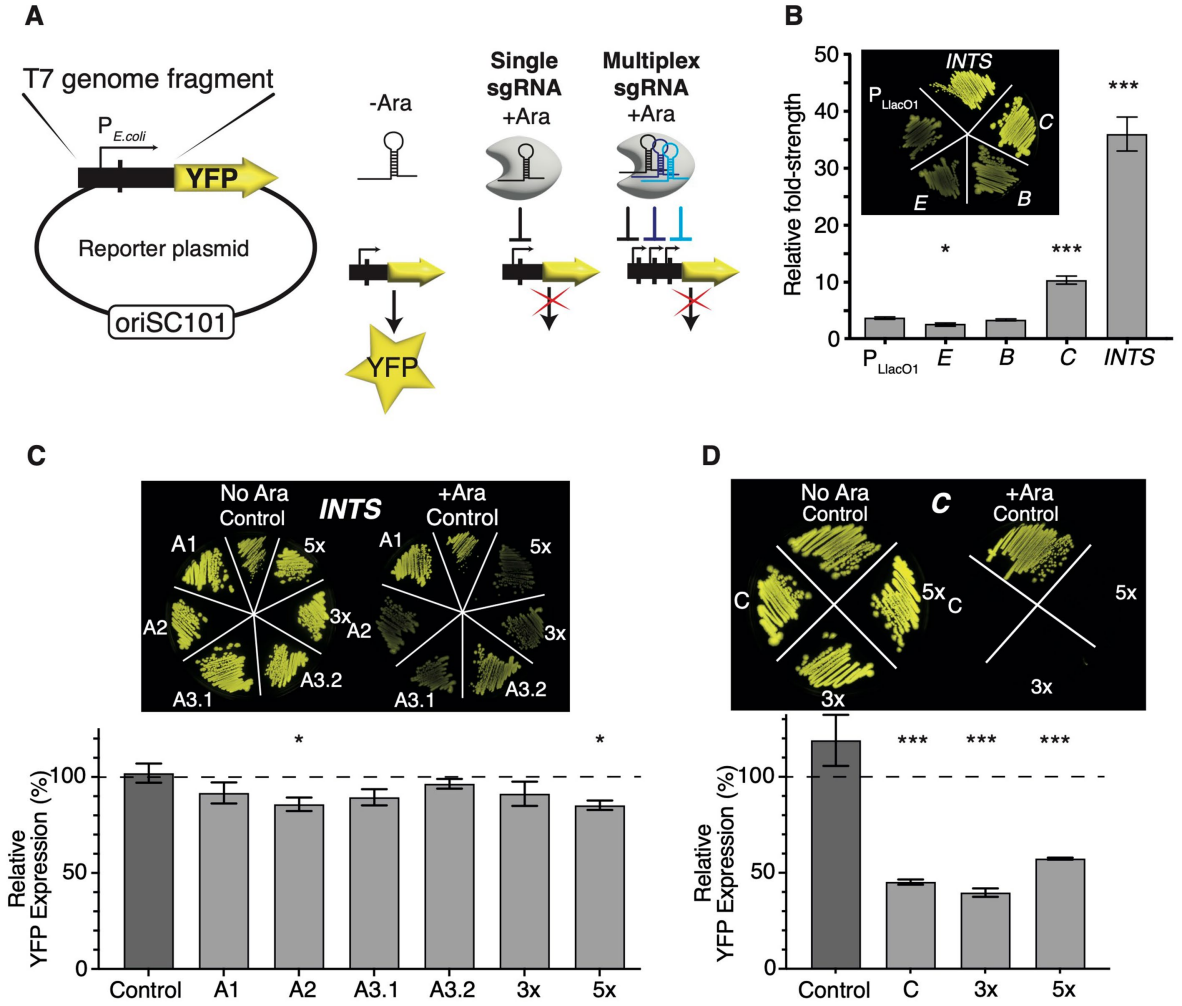
Quantifying the efficacy of CRISPRi using fluorescent reporters. **(A)** Construction of fluorescent *E. coli* RNAP phage promoter reporter plasmids. T7 genome fragments encoding selected *E. coli* RNAP phage promoters were cloned into an oriSC101 plasmid [pZS1 (Lutz and Bujard, 1997)] in front of a *yfp* Venus open reading frame which carries its own ribosome binding site (not shown). Transcriptional activation of promoters leads to the production of YFP Venus. Targeting promoters with CRISPRi by supplying the inducer L-arabinose and single or multiplex sgRNAs allows us to quantify the efficacy of knockdown by measuring YFP fluorescence levels. **(B)** Comparison of *E. coli* RNAP phage promoter strengths. YFP expression levels were normalised to uninduced pZS12-YFP Venus and showed fold-change promoter strength. Insert: fluorescence image of TB549 with promoter plasmids and uninduced pZS12-YFP Venus, which is relatively leaky in TB549. *p* < 10^−3^ in a nonparametric t-test comparing induced pZS12-YFP to *C* or *INTS* indicated by ***, and 0.02 for *E* indicated by *. *N* = 4 for all data shown. **(C)** Fluorescence knockdown efficiency across *INTS* or **(D)** *C* (T7 RNAP) using single, 3× or 5× multiplex sgRNAs. The inserts show fluorescence images of TB549 with corresponding sgRNA, and fluorescent reporter plasmids streaked on selective LB agar plates without (left) or with L-arabinose (right). The graphs show fluorescence knockdown efficiency normalised to controls lacking L-arabinose and dCas9 expression. The y-axis shows % YFP expression to uninduced controls lacking L-arabinose. In **(C)**, *p* < 0.05 in a pairwise t-test comparing Control sgRNA with A2 or 5× sgRNA indicated by *. For **(D)**, *p* < 10^−3^ in a pairwise t-test of C, 3× or 5× to the control sgRNA indicated by ***. *N* = 6 for all data shown. Error bars are one standard error of the mean. Please note that the inset fluorescent images in panels **(C)** and **(D)** serve as a non-quantitative guide for visual comparison of fluorescent knockdown effects. The brightness and contrast values of inset images **(C,D)** have been adjusted on a linear scale across identical values using ImageJ.

Next, I constructed fluorescent transcriptional reporter plasmids to assay the expression strength of *E. coli* RNAP-dependent promoters encoded by T7 phage, and to quantify the efficacy of transcriptional repression by CRISPRi by fluorescence knockdown measurements (Figure 2A). I built reporter plasmids following a previously published approach (Zaslaver et al., 2006) by placing T7 DNA fragments containing the desired promoters flanked by ~150 bp up-and downstream sequences onto a low copy oriSC101 plasmid (Lutz and Bujard, 1997) upstream of a yellow fluorescent protein gene (*yfp* Venus). The *yfp* ORF is preceded by a strong ribosome binding site [RBS (Lutz and Bujard, 1997)], and YFP expression levels report on transcript abundance and transcriptional activity of the promoters of interest. I included all annotated *E. coli* RNAP-dependent T7 phage promoters, *B*, *C*, and *E*, and a ~570 bp fragment of *INTS* containing promoters *A1*, *A2*, and *A3* (nucleotides 439–1,012, omitting promoter phiOL) to compare their relative strengths and to validate the target choice.

The reporter plasmids had mild to strong detrimental effects on the growth rates of their host strains, which corroborates previous findings that T7 phage genome fragments cloned into plasmids can be detrimental or even toxic to growth (Studier and Rosenberg, 1981). Notably, *INTS* turned out to be highly toxic and showed a heterogeneous small colony phenotype, and an unusual bi-phasic growth pattern (Supplementary Figure 2). A large colony that displayed uniform growth patterns while expressing very high levels of YFP was selected at random. This reporter plasmid had a small deletion upstream of the *go0.3* fragment (but outside of promoter *A3* and the sgRNA binding site) that was included in the *INTS* construct (Supplementary Figure 2). This stabilised promoter plasmid was used for all experiments shown in this study hereafter.

Assaying YFP expression as a proxy of promoter strength enabled a ranking of *E. coli* RNAP promoters. *E* and *B* were the weakest promoters and slightly weaker or on par with an IPTG-induced P_LlacO1_-YFP control plasmid, while promoter *C* and the *INTS* fragment showed a ~3-fold and ~10-fold higher promoter strength than P_LlacO1_-YFP (Figure 2B). Promoters *E* and *B* are described as minor *E. coli* promoters (Studier and Rosenberg, 1981) and their role in T7 infection remains unclear. Promoters *E* and *B* were consequently excluded from further investigation and analysis that was focused on *INTS* and *C (gp1)*, apart from targeting *B (gp0.5)* with the 5× multiplex sgRNA.

TB549 was then sequentially transformed with sgRNA plasmids and fluorescent reporter plasmids, and first, the effects of targeting *A1*, *A2*, and *A3* within *INTS* with individual sgRNAs A1, A2, A3.1, and A3.2 were evaluated for cultures growing at 37°C. The overall efficacy of CRISPRi using single sgRNAs was low, resulting in a ~15% decrease in YFP expression at best (Figure 2C). Nevertheless, the order of targeting *A1*, *A2*, and *A3* promoters on *INTS* appeared to be important: targeting *A1* or *A3* with a sgRNA binding inside the *ocr* fragment (sgRNA A1 or A3.2) showed very weak or no discernible effects on fluorescence, while targeting *A2* and *A3* (sgRNA A2 or A3.1) had mild, and in the case of A2, significant effects (Figure 2C). In contrast, targeting the T7 RNAP reporter driven by *C* was very efficient, leading to a ~60% reduction in the expression of fluorescence (Figure 2D). Supplementary Figure 3 shows the non-normalised values of the fluorescence knockdown experiments as a reference.

Subsequently, I constructed and tested arrays of 3× and 5× multiplex sgRNAs which contained A3.1, A3.2, and C sgRNAs for the 3× multiplex array, and A2, A3.2, B, and two sgRNAs targeting *C* for the 5× multiplex array. Here, the rationale was that targeting *INTS* at several locations may improve knockdown due to the presence of several roadblocks, or by blocking RNAP access to alternative promoters. Targeting *C* twice and additionally *B* at the same time may facilitate additive or even synergistic effects towards blocking the T7 phage lifecycle in later experiments using the same sgRNA constructs. I found that the 5× array showed only a mild but significant improvement in efficacy while the 3× array showed no further improvement in knocking down YFP expression from *INTS* (Figure 2C). Surprisingly, while the 3× sgRNA array was targeting *C* with similar efficacy as the single sgRNA, targeting *C* at two positions simultaneously using the 5× sgRNA had weakly antagonistic effects on YFP knockdown (Figure 2D).

Taken together, these data suggest that there are weak position-dependent polar effects across the *INTS* promoter array: blocking *A1* had no significant effects on YFP expression, likely as *E. coli* RNAP can initiate transcription at promoters *A2* and *A3*. In turn, blocking *A2* or *A3* lowered YFP expression, likely by limiting the ability of *E. coli* RNAP to initiate transcription downstream at one or multiple alternative promoters. Blocking *E. coli* RNAP even further downstream of *A3* within the *gp0.3* fragment (sgRNA A3.2) had no effect on YFP expression. One possibility is that the very high strength of the promoter array of *INTS* limits the efficacy of CRISPRi to operate as an efficient roadblock of transcription elongation outside of the promoter array. Also, these results show that repeatedly and simultaneously targeting the same region does not necessarily lead to additive or synergistic knockdown effects.

### Efficacy of CRISPRi towards T7 phage during infection

**Figure 3:**
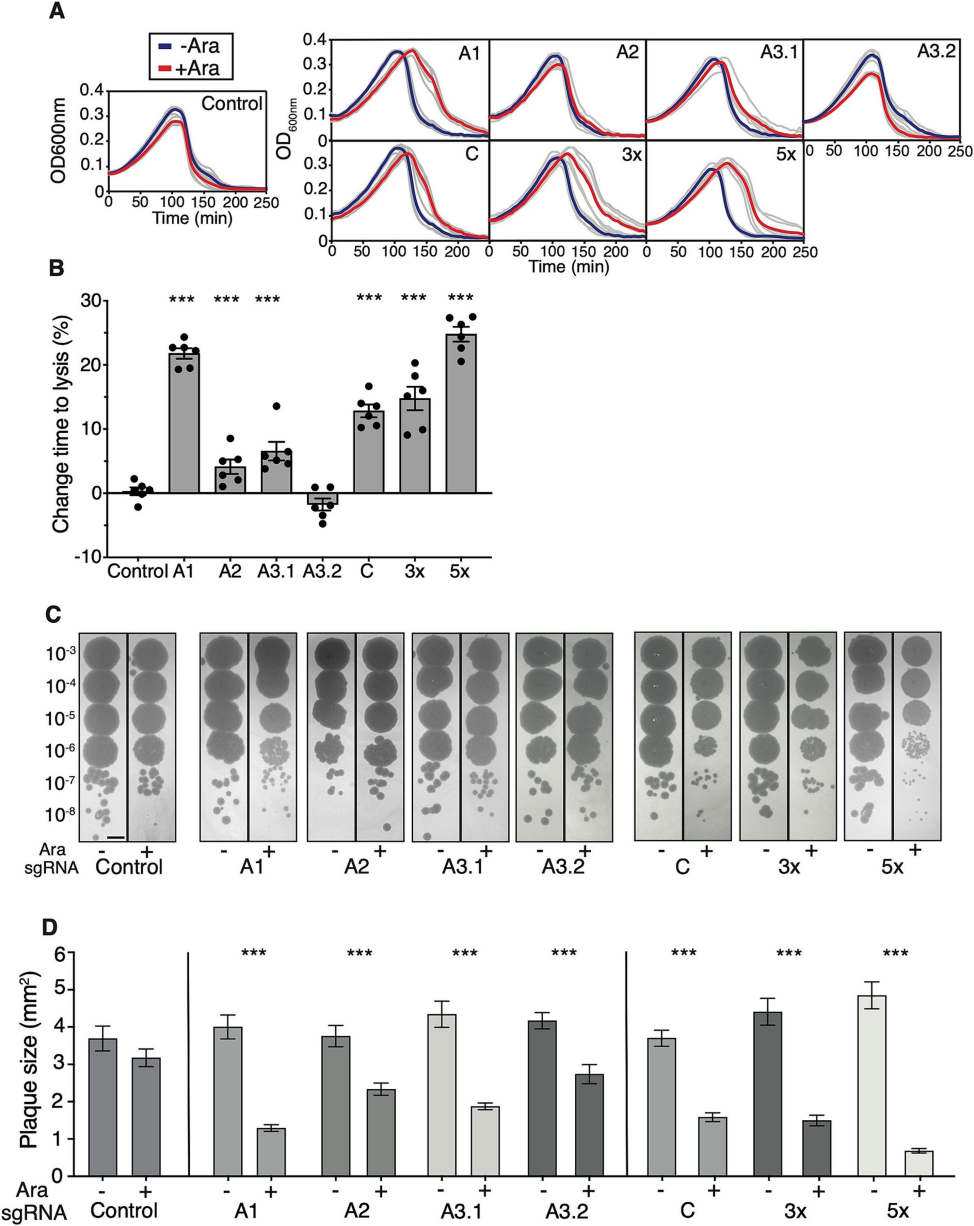
Effects of CRISPRi on the time to lysis and plaque size of T7 phage. **(A)** Effects of CRISPRi expression on culture lysis. Growth curves of cultures are shown infected with T7 phage at MOI 10^−5^ with (red) or without (blue) 0.1% L-arabinose. Grey lines are raw data, and thick lines are means (*n* = 6). Left: control sgRNA. Top row: individual sgRNAs targeting *INTS*. Bottom row: individual sgRNA targeting *C*, and 3× and 5× multiplex constructs targeting several T7 phage regions simultaneously. **(B)** Quantification of the time to lysis of T7 phage. The onset of culture lysis was quantified by finding the inflection points of lysis curves using a procedure described in Material and Methods. The inflection time points of L-arabinose-induced cultures were normalised to their respective controls lacking L-arabinose. Two-tailed t-test comparing the Control sgRNA to A3.2 sgRNA shows no significance, all other paired t-tests with Control sgRNA show significance with *p* < 10^−3^ indicated by ***. **(C)** Effects of CRISPRi expression on the efficiency of plaquing (EOP). Expression of CRISPRi has noticeable effects on plaque size. Shown are regions of interest of equal size, scale bar = 5 mm. The quantification of EOP is shown in Supplementary Figure 3. **(D)** Quantification of plaque size shows the effects of CRISPRi. Shown are data from three independent experiments, and a minimum of three plaques were analysed per replicate. Error bars are one standard error of the mean. *p* < 10^−3^ using a two-tailed t-test indicated by ***. Numbers of plaques per sgRNA (−/+ L-arabinose): Control 20/15, A1 19/22, A2 17/15, A3.1 13/23, A3.2 15/13, C 16/22, 3× 16/27, 5× 11/28. Error bars are one standard error of the mean.

Next, I sought to examine the ability of CRISPRi to interfere with the time to lysis of bacterial cultures by T7 phage which can be used as a robust proxy for the resilience of the host towards phage infection (Chitboonthavisuk et al., 2022). To achieve this, I compared the onset of lysis of bacterial cultures expressing sgRNAs and dCas9 with those lacking the dCas9 inducer L-arabinose at identical multiplicities of infection (MOI) and initial bacterial cell densities. To increase the contrast between the onset of lysis between induced and uninduced cultures, all lysis experiments were performed at 30°C which extends the eclipse period of T7 phage from ~10 min to ~17 min (Calendar, 2006). I grew replicates of cultures with or without L-arabinose to the late exponential phase, diluted them into fresh LB broth, and added small amounts of T7 phage (MOI 10^−5^). The addition of L-arabinose and dCas9 expression led to a notable increase in time-to-lysis of cultures expressing A1 and C sgRNA and the 3× and 5× multiplex constructs (Figure 3A). The onset of culture lysis was then quantified by determining the inflection point of the growth curve at which population collapse by lysis occurred, and at which the slope of the growth curve turned negative (Material and Methods). This showed that the effects of CRISPRi targeting *INTS* followed a slightly different order compared to fluorescent knockdown assays: among all individual sgRNAs targeting *INTS*, A1 had the strongest effect on the time to lysis, which increased by ~22%. The other *INTS* targets followed a similar trend observed in the fluorescent reporter assays: targeting A2 and A3.1 had mild but significant effects while targeting A3.2 at the very end of *INTS* had no effect (Figure 3B) on the time to lysis. Overall, this order of effects approximately follows the chronological order of appearance of the *INTS* DNA segment into the cell during capsid-catalysed T7 DNA ejection, with *A1* appearing first, *A2* second, and so on. There are two interesting consequences for the DNA ejection model arising from this observation: one possibility is that *A1* and upstream sequences are important for RNAP binding or loading and initiation of RNAP-catalysed DNA internalisation, and that dCas9 binding close to *A1* is interfering with this process. Another possibility is that the early virion-catalysed T7 DNA ejection process partially depends on the host’s RNAP. This assumption contradicts the findings of the experimentally established model of RNAP-independent and entirely virion-catalysedT7 DNA ejection (Garcia and Molineux, 1995).

Surprisingly, while I found that transcription from *C* can be knocked down very efficiently at the fluorescent reporter level, I observed only a moderate effect on the time to lysis when targeting T7 RNAP during infection using sgRNA C. Similar effects were documented in a recent study that used CRISPRi-Art to target phage mRNA rather than DNA (Adler et al., 2023). Targeting the T7 phage with the 3× multiplex array sgRNA showed a slight improvement over targeting *C* only, thereby suggesting a weakly positive combinatorial effect of targeting *INTS* and *C* simultaneously. The 5× multiplex sgRNA array displayed the strongest effect and a marked ~25% increase of the time to lysis, and a relatively strong combinatorial effect of blocking several *E. coli* RNAP-dependent promoters at once (Figure 3B).

Finally, EOP, a measure of infection productivity of the phage, remained unaltered across all sgRNAs (Figure 3C and Supplementary Figure 4), but there were moderate to strong effects on plaque size. To prevent the fusion of plaques and to allow quantification of plaque size using the drop dilution method, top agar plates were incubated at 22°C. Here, the order of effects followed the results of the time-to-lysis experiments. Blocking *A2*, *A3.1*, and *A3.2* showed marked effects while blocking *A1* had the strongest effect, leading to a ~3-fold decrease in plaque size. Also, dCas9 knockdown of T7 RNAP using a single sgRNA had moderate effects on plaque size reduction, thus following the trend observed in the time-to-lysis experiments. Again, the 5× multiplex approach was found very efficient leading to a ~8-fold reduction in plaque size (Figure 3D). These results show that while dCas9 causes imperfect blockage or knockdown of T7 during infection, there is a substantial additive effect when using multiplex sgRNAs. Furthermore, while dCas9 cannot mediate resistance to T7 phage infection or affect EOP, it can interfere with T7 phage during the infection process, significantly increase the time-to-lysis, and decrease plaque size.

## Discussion

CRISPRi approaches based on *S. pyogenes* dCas9 have been highlighted in the past decade as highly flexible tools to study gene function across many different microbial species (Qi et al., 2013; Peters et al., 2016; Liu et al., 2017; Lee et al., 2019; Peters et al., 2019; Mutalik et al., 2020; Rishi et al., 2020; Liu et al., 2021), and to construct synthetic gene regulatory circuitry (Kuo et al., 2020; Rueff et al., 2023). Until recently, there was little evidence of the usability of CRISPRi or the popular *S. pyogenes* dCas9-dependent CRISPRi to study phage gene function or to tune phage lifecycles, although three reports suggest that phages or selected phage targets are amendable to *S. pyogenes* dCas9 knockdown (Rousset et al., 2018; Widom et al., 2019; Barman et al., 2022). In the current research, I explored the ability of CRISPRi to interfere with the lifecycle of T7 phage by specifically targeting *E. coli* RNAP-dependent phage promoters which are essential for DNA internalisation and lifecycle execution (Calendar, 2006). By using a limited set of sgRNAs and two multiplex constructs, the present research is a proof-of-concept study that probes the ability of dCas9 to interfere with the lifecycle of T7 phage.

I built a set of T7 phage fluorescent reporters to measure the strength of *E. coli* RNAP phage promoters annotated on the T7 genome and established a clear order of strength across these promoters. Promoter *C* and the promoter array within *INTS* are exceptionally strong, and the promoter array is three times stronger than *C*. The strength of the minor promoters *B* and *E* was comparable to IPTG-induced P_LlacO1_, which is a synthetic promoter based on the strong phage Lambda P_L_ promoter (Lutz and Bujard, 1997). Furthermore, *INTS* has a substantial detrimental effect on growth (Supplementary Figure 2) that stems either from unconstrained expression of YFP, or expression of a *gp0.3* fragment that is included in the fluorescent reporter construct and that may be toxic at high expression levels. The stabilised *INTS* reporter displayed a 45 bp deletion upstream of the *gp0.3* fragment which truncates the 5′ end of the 0.3 mRNA fragment including the putative RBS of the *gp0.3* fragment, suggesting that this fragment is in fact detrimental to growth. The stabilised *INTS* reporter plasmid also had a measurable detrimental effect on growth which could be based on YFP overexpression or excess titration of host RNAP from the limited cellular pool (Supplementary Figure 2). Nevertheless, this raises the question of whether the early steps in the infection process starting with host RNAP binding and early gene transcription lead to an immediate effect on cell growth by either titration of RNAP or by expression of toxic early gene products. A recent report studying T7 phage infection at the single-cell level does not support this notion but shows that host cell growth stalls within the first 2–3 min of phage genome incorporation into the cell (Wedd et al., 2024).

Probing the promoter array within *INTS* with individual sgRNAs showed that there is no dominant promoter within this region as blocking *A1* had very weak or no effect on YFP fluorescence knockdown. This finding suggests that *E. coli* RNAP can initiate expression from the *A2* and *A3* promoters downstream of the roadblock. Concurrently, the strongest knockdown effects were apparent when either *A2* or *A3* were targeted, implying that initiation of transcription is less efficient downstream of *A2* or *A3* and that dCas9 roadblock at these positions had polar effects on transcription. Placing another sgRNA (A3.2) further downstream into the beginning of the *ocr* coding region had no effect at all. One explanation is that the knockdown efficacy of CRISPRi is inversely proportional to promoter strength (knockdown efficacy decreases with increasing promoter strength), a factor that may explain the low efficacy of targeting *INTS* in general. More specifically, the high frequency of head-on collisions between active transcription bubbles and bound dCas9 may decrease CRISPRi knockdown efficacy, although this has not been investigated in greater depth. Another possibility is that sgRNAs A2, A3.1, and A3.2 were less efficient by design and binding location within *INTS*, which is a factor that can lead to variable yet difficult-to-predict knockdown outcomes (Yu et al., 2024). Nevertheless, other work has shown that the overall efficacy of targeting promoters outside of the transcription start to bubble or downstream of the −35/−10 region is highly efficient (Rishi et al., 2020).

Another factor that could reduce CRISPRi efficacy [which has been reported to be at 90–99% knockdown efficiency (Qi et al., 2013; Peters et al., 2019)] is that the fluorescence knockdown targets are located on an oriSC101 plasmid that has about four copies/cell (Jahn et al., 2016). For instance, sgRNA-loaded dCas9 may be limiting within the cell, and targeting plasmids abound in multiple copies becomes less efficient due to the limited availability of sgRNA-dCas9 complexes. This is supported by the results shown in Supplementary Figure 5, and the knockdown of a single copy target integrated into the host genome shows greater effects (~65% YFP expression reduction). I also found that the apparent knockdown efficacy depends on the type of growth medium used (~80% YFP expression reduction in M9 Glycerol; Supplementary Figure 5). Here, I speculate that the high autofluorescence of LB broth may interfere with the detection of the YFP fluorescence signal, leading to apparently lower knockdown efficacy. Of note, other studies reporting higher knockdown efficacies have not only targeted single-copy and chromosomally inserted targets (Qi et al., 2013; Peters et al., 2019), but also used flow cytometry for the detection of fluorescence which greatly exceeds the sensitivity of commonly used microtiter plate readers.

The results also show that *E. coli* RNAP promoter *C* in front of T7 RNAP is very strong, but that it can be knocked down with relatively high efficiency when targeting the 5′-end of *gp1*. It can also be speculated that, due to its high strength, promoter *C* may act together with the internalisation signal to facilitate internalisation (which is suggested to be entirely *INTS*-dependent), or at least aid internalisation of mutant phages lacking *A1*, *A2*, and *A3* that are able to infect host cells while (Garcia and Molineux, 1996). In turn, promoter *B* which has been implicated as an accessory promoter for internalisation is ~3× weaker than *C* and only mildly stronger than P_LlacO1_.

Transcriptional fluorescent reporters have been extensively used to study differential gene regulation and transcription in *E. coli* or *Salmonella typhimurium* (Zaslaver et al., 2006; Freed et al., 2008; Silander et al., 2012). Similar studies have not been conducted using phage-derived *E. coli* promoters, possibly due to the unavailability of an appropriate phage *E. coli* promoter collection. The promoter plasmids constructed for the work presented here can be used in future studies that explore their activity in different environments. This can include testing a variety of carbon sources and growth conditions, different ecological contexts such as mixed species cultures or biofilms that may influence promoter expression and subsequently affect the lifecycle and infectivity of T7 phage under these likely more natural conditions.

Surprisingly, the results presented here show that blocking the first strong promoter *A1* on *INTS* had strong effects on the time to lysis and infectivity of the phage, but not the expression of the fluorescent reporter construct. This suggests that the chronological appearance of T7 phage DNA in the cell affects the efficacy of CRISPRi towards T7. Promoter *A1* is located on the left-most leading end of the linear T7 phage genome, which is the first section of T7 DNA to appear within the host cell. Nevertheless, this result is surprising as a suite of elegant experiments has established that the internalisation signal is ejected independent of *E. coli* RNAP or T7 RNAP activity into the cell through a virion-catalysed step, and that it was thought to be initially inaccessible to host proteins (Moffatt and Studier, 1988; Garcia and Molineux, 1995, 1996). This is based on the observation that T7 DNA is not degraded by host restriction endonucleases during the first few minutes after appearing within the cell (Moffatt and Studier, 1988). Furthermore, a GATC motif located in the T7 early region that is supposed to be rapidly methylated by DNA adenine methyltransferase (Dam) upon T7 genome entry remains unmethylated for extended time spans (Garcia and Molineux, 1995).

One possible explanation for the results shown in the current work here is that sequences upstream of *A1* are required to load *E. coli* RNAP, and blocking *A1* with CRISPRi interferes with this process. Thus, *A1* may play a dominant role during the RNAP-dependent phase of the T7 genome internalisation process that cannot be fully compensated for by *A2* or *A3*. Another possibility is that A2, A3.1, and A3.2 sgRNAs are simply less efficient in interfering with T7 DNA internalisation based on design, although A2 and A3.1 were able to knock down the expression of the fluorescent reporter. Previous work has shown that sgRNAs targeting the same genomic feature can have varying effects (Rishi et al., 2020), but the reasons for sgRNA-based variability in knockdown efficiency, other than effects caused by the “bad seed” effect (Rostain et al., 2023), are difficult to predict (Cui et al., 2018; Yu et al., 2024).

Two recent reports have revealed that both temperate phages *λ* and P1 are amenable to knockdown by dCas12a (Piya et al., 2023) and phages T4 and T7 to dRfxCas13d or CRISPRi-ART (Adler et al., 2023). While dCas12a has a somewhat limited target range due to its relatively large and T-rich PAM (TTTV), dRfxCas13d targets transcripts and may find broad use as a tool to interrogate both phage and bacterial gene function. Overall, although *S. pyogenes* dCas9-dependent CRISPRi is less efficient than dCas12a, it offers greater flexibility due to its NGG PAM and usability to study knockdown effects and combinatorial effects, even if it does not completely abolish T7 infectivity. Also, previous studies that have aimed at controlling the lifecycle of T7 through an “infectivity switch” rely on phage engineering (Chitboonthavisuk et al., 2022). The present study shows that similar, though milder effects on the phage’s time to lysis, can be achieved by rational programming of dCas9 with sgRNAs instead of genetic engineering of the phage.

Further research into specifically and selectively targeting lifecycle features of phages using dCas12a or CRISPRi-ART can open new avenues for rational engineering and programming of otherwise lytic bacteriophages for synthetic biology applications. These could include the engineering of synthetic lysis-lysogeny switches to control otherwise obligatorily lytic phages. Synthetic lifecycle switches can be useful tools for the propagation and production of lytic phages at scale to minimise unwanted phage-host coevolution that is unavoidable during culture lysis (Perry et al., 2015). Other avenues could explore engineering synthetic communication between phages [a natural feature of Gram-positive phages (Stokar-Avihail et al., 2019)] or phages and their hosts by regulating the transfer and expression of sgRNAs and dCas9 for reciprocal or unidirectional programmed gene regulation and knockdown. Overall, CRISPRi has proven to be an invaluable tool for studying biological processes. Exploring future avenues in phage engineering and control could further expand its applications within the fields of synthetic biology and engineering biology.

## Data Availability

The datasets presented in this study can be found in online repositories. The names of the repository/repositories and accession number(s) can be found at: https://www.biorxiv.org/content/10.1101/2024.05.15.594216v1.supplementary-material, BIORXIV/2024/594216. Data is available here: 10.5281/zenodo.14714789.

## Supplementary material

SUPPLEMENTARY FIGURE 1 Growth rates of TB549 expressing sgRNAs in the presence or absence of dCas9 induction. Shown are doubling times in minutes of TB549 expressing sgRNAs in the absence or presence of dCas9 induced with 0.1% L-arabinose. There is no statistical significance between uninduced and induced dCas9 expression for all sgRNAs, or TB549 alone (two-tailed t-tests). *N* = 6 for all bars shown. Error bars are one standard error of the mean.

SUPPLEMENTARY FIGURE 2 Growth rates of strains expressing fluorescent promoter reporters. **(A)** Doubling times of BW25113 expressing respective fluorescent reporter plasmids or pZS12-YFP Venus induced with 500μM IPTG. All fluorescent reporters increase doubling time compared to BW25113 (pairwise t-test *p* < 10^−3^ indicated by ***). *N* = 4 for all data shown. **(B)** Growth curve of the pZS1-*INTS*-YFP “small colony” variant in TB549 in the absence of dCas9 expression. Indicated are the two growth phases that appear to be separated by partial culture lysis. **(C)** Growth curve of the pZS1-*INTS*-YFP “big colony” variant. **(D)** Doubling times of growth phases 1 and 2 of pZS1-*INTS*-YFP “small colony” and pZS1-*INTS*-YFP “big colony” variants. **(E)** Sanger sequencing chromatograms showing a 45 bp deletion in the pZS1-*INTS*-YFP “big colony” reporter plasmid and the original “small colony” variant sequence. *N* = 6 for growth curves. Bold line shows the mean, shaded curve is one standard error of the mean. Error bars are one standard error of the mean.

SUPPLEMENTARY FIGURE 3 Raw data fluorescence knockdown experiments. Shown is the raw data of fluorescence knockdown experiments of *INTS*-YFP and *C*-YFP fluorescent reporters as fluorescence normalised by optical density at 600 nm (Fluorescence/OD_600nm_). **(A)** Knockdown efficiency of *INTS*-YFP, and **(B)** knockdown efficiency of *C*-YFP by indicated sgRNAs and multiplex sgRNAs. *p* < 10^−3^ using a two-tailed t-test indicated by ***, *p* < 0.05 indicated by * between induced and uninduced dCas9 controls, respectively.

SUPPLEMENTARY FIGURE 4 Efficiency of plaquing (EOP) of phage T7 on strains expressing sgRNAs. Shown are EOPs of phage T7 growing on TB549 top agar in the absence or presence of the dCas9-inducer L-arabinose (0.1%). There is no statistical significance between uninduced and induced dCas9 expression on EOP (two-tailed t-tests). The means of three independent experiments are shown. Error bars are one standard error of the mean.

SUPPLEMENTARY FIGURE 5 CRISPRi knockdown efficacy of a single-copy target and at different dCas9 induction levels. **(A)** CRISPRi knockdown efficacy when targeting a single copy and constitutively expressed YFP. Shown is knockdown or PR-YFP inserted in *att*P21 in strain TB566 carrying a very low copy pZS*1-P_araBAD_-dCas9 plasmid induced with 0.1% arabinose using two different sgRNAs, YFPI and YFPII. Apparent knockdown efficiency in M9 glycerol is near 80%, and about 65% in LB broth. T-tests between indicated pairs are highly significant (*p* < 0.005). **(B)** Knockdown efficacy of promoter *C* across 0.01, 0.05 and 0.1% arabinose show no significant changes in YFP fluorescence levels.
