# Supplementary figures and images for: Programming CRISPRi to control the lifecycle of bacteriophage T7
Source: Front Microbiol. 2025 Feb 12;16:1497650. doi: 10.3389/fmicb.2025.1497650 (PMC11863960; doi:10.3389/fmicb.2025.1497650)

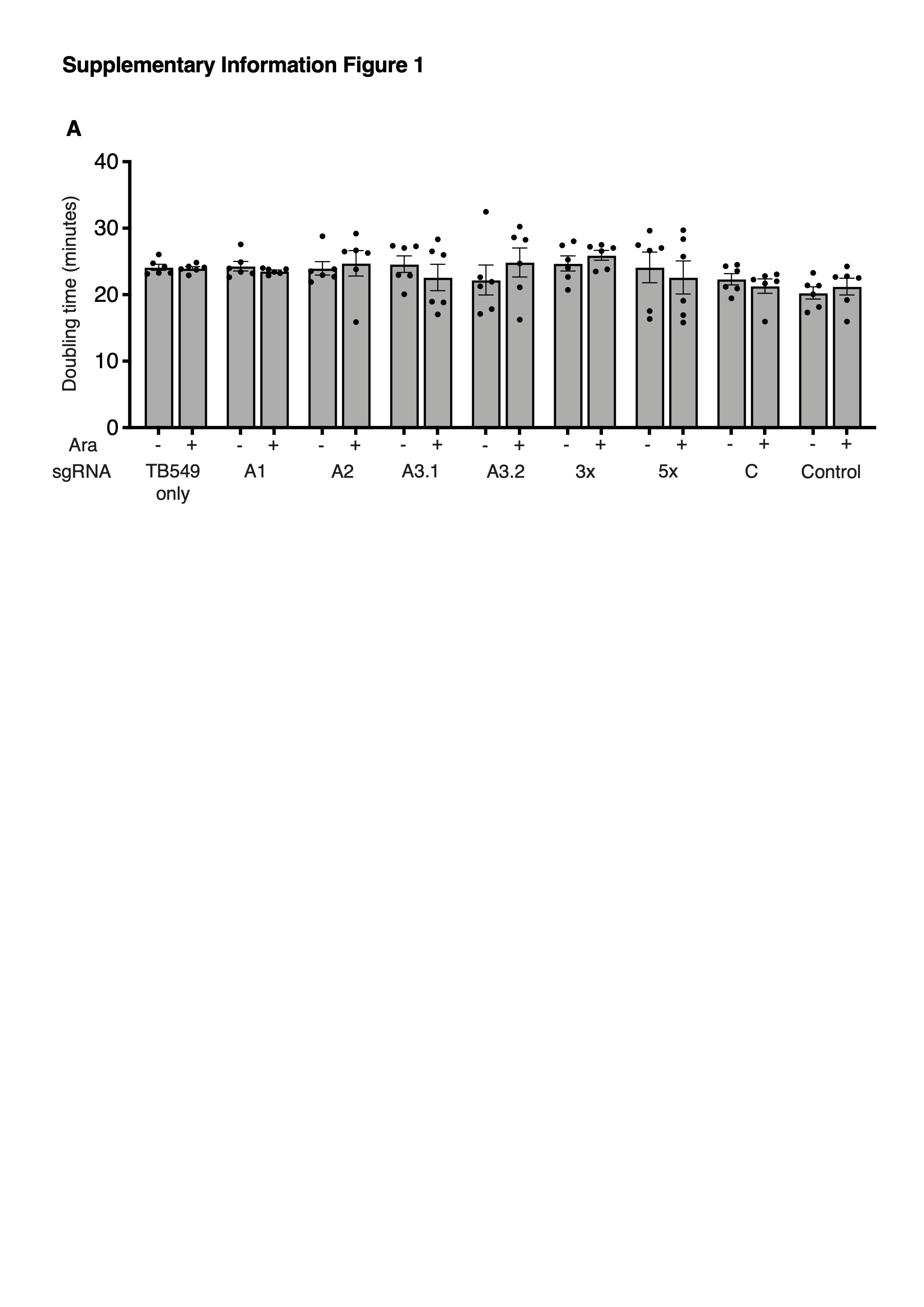

Supplement: SUPPLEMENTARY FIGURE 1 — Growth rates of TB549 expressing sgRNAs in the presence or absence of dCas9 induction. Shown are doubling times in minutes of TB549 expressing sgRNAs in the absence or presence of dCas9 induced with 0.1% L-arabinose. There is no statistical significance between uninduced and induced dCas9 expression for all sgRNAs, or TB549 alone (two-tailed t-tests). N = 6 for all bars shown. Error bars are one standard error of the mean. [file Image_1.jpeg]

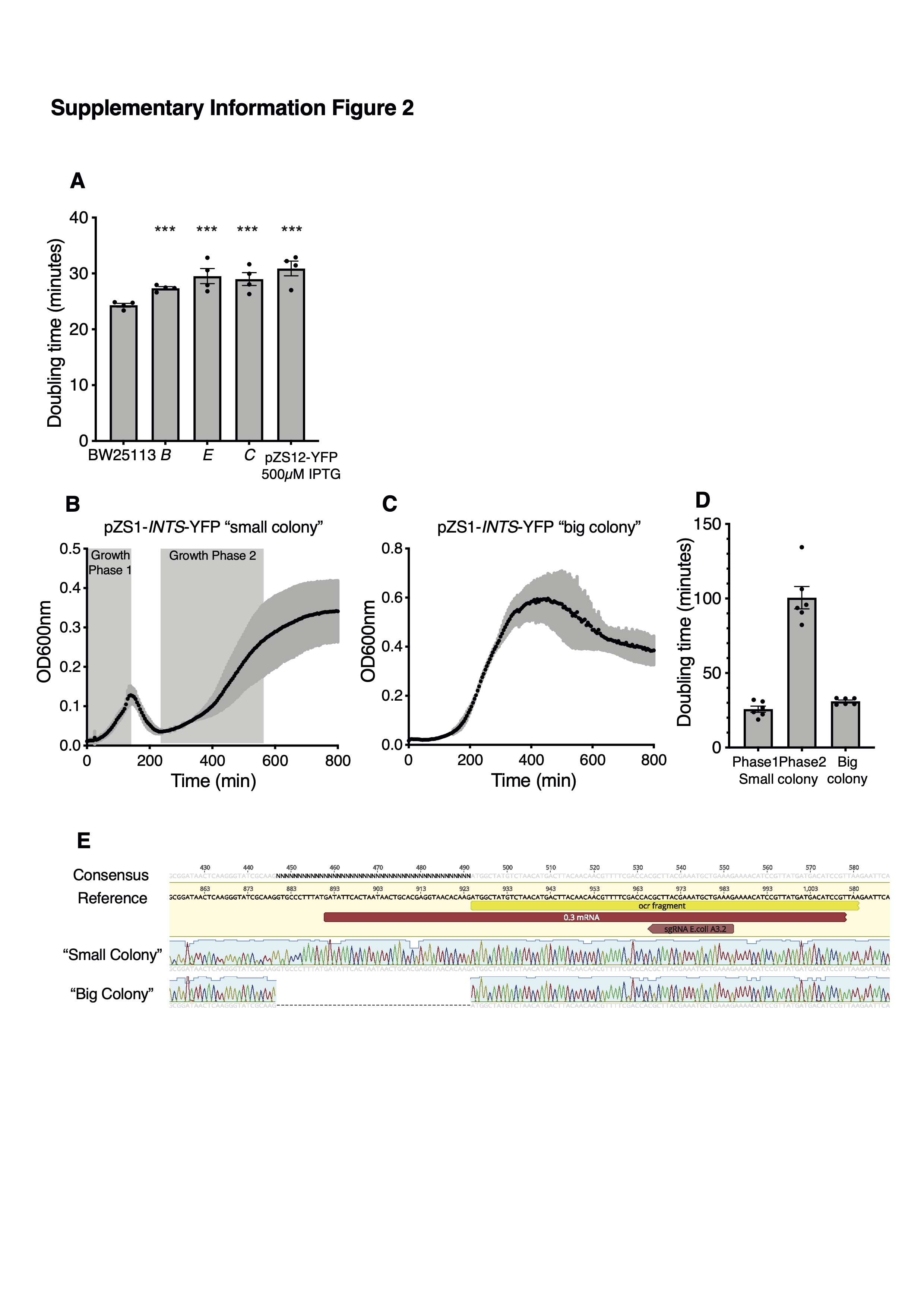

Supplement: SUPPLEMENTARY FIGURE 2 — Growth rates of strains expressing fluorescent promoter reporters. (A) Doubling times of BW25113 expressing respective fluorescent reporter plasmids or pZS12-YFP Venus induced with 500μM IPTG. All fluorescent reporters increase doubling time compared to BW25113 (pairwise t-test p < 10−3 indicated by ***). N = 4 for all data shown. (B) Growth curve of the pZS1-INTS-YFP “small colony” variant in TB549 in the absence of dCas9 expression. Indicated are the two growth phases that appear to be separated by partial culture lysis. (C) Growth curve of the pZS1-INTS-YFP “big colony” variant. (D) Doubling times of growth phases 1 and 2 of pZS1-INTS-YFP “small colony” and pZS1-INTS-YFP “big colony” variants. (E) Sanger sequencing chromatograms showing a 45 bp deletion in the pZS1-INTS-YFP “big colony” reporter plasmid and the original “small colony” variant sequence. N = 6 for growth curves. Bold line shows the mean, shaded curve is one standard error of the mean. Error bars are one standard error of the mean. [file Image_2.jpeg]

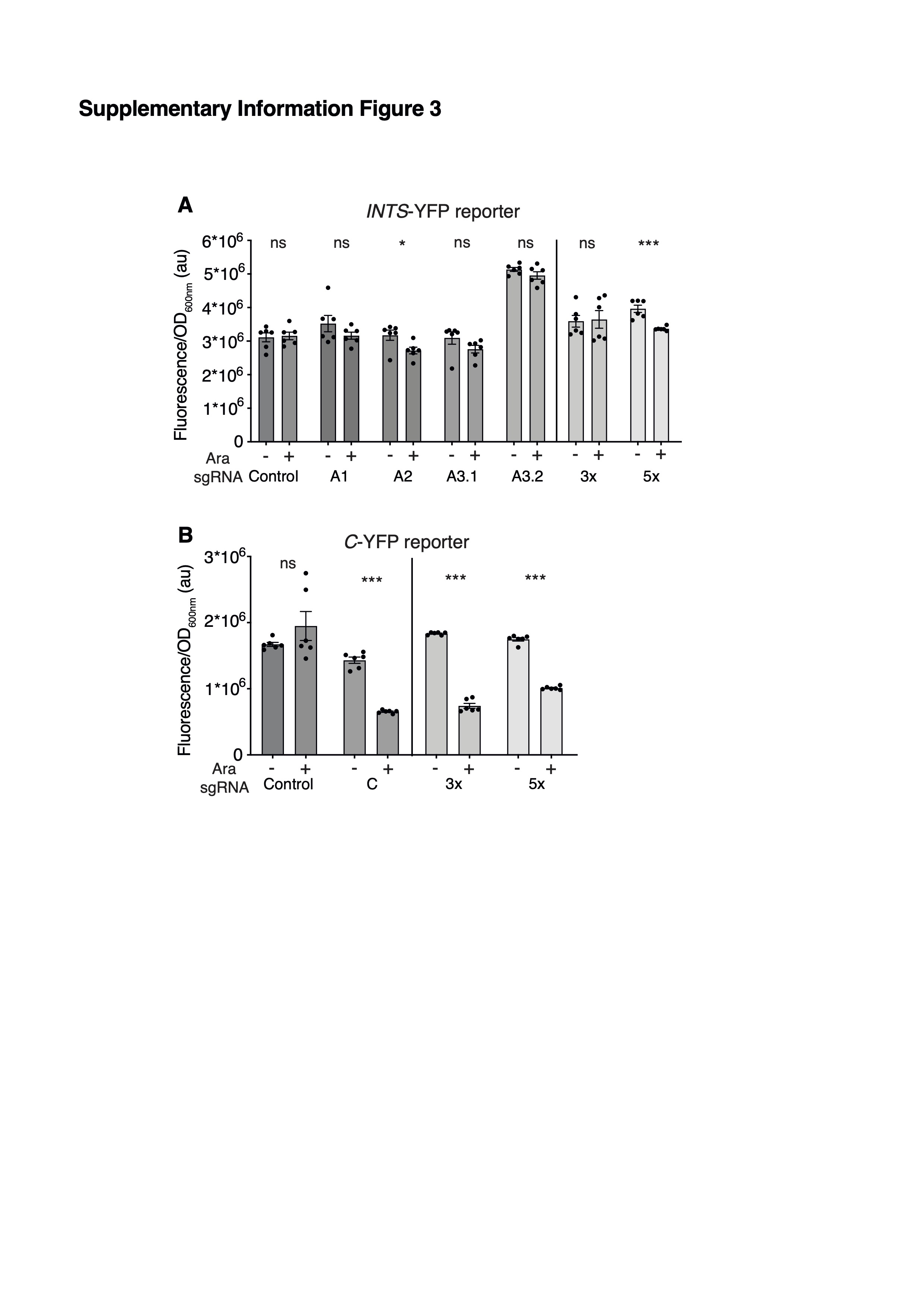

Supplement: SUPPLEMENTARY FIGURE 3 — Raw data fluorescence knockdown experiments. Shown is the raw data of fluorescence knockdown experiments of INTS-YFP and C-YFP fluorescent reporters as fluorescence normalised by optical density at 600 nm (Fluorescence/OD600nm). (A) Knockdown efficiency of INTS-YFP, and (B) knockdown efficiency of C-YFP by indicated sgRNAs and multiplex sgRNAs. p < 10−3 using a two-tailed t-test indicated by ***, p < 0.05 indicated by * between induced and uninduced dCas9 controls, respectively. [file Image_3.jpeg]

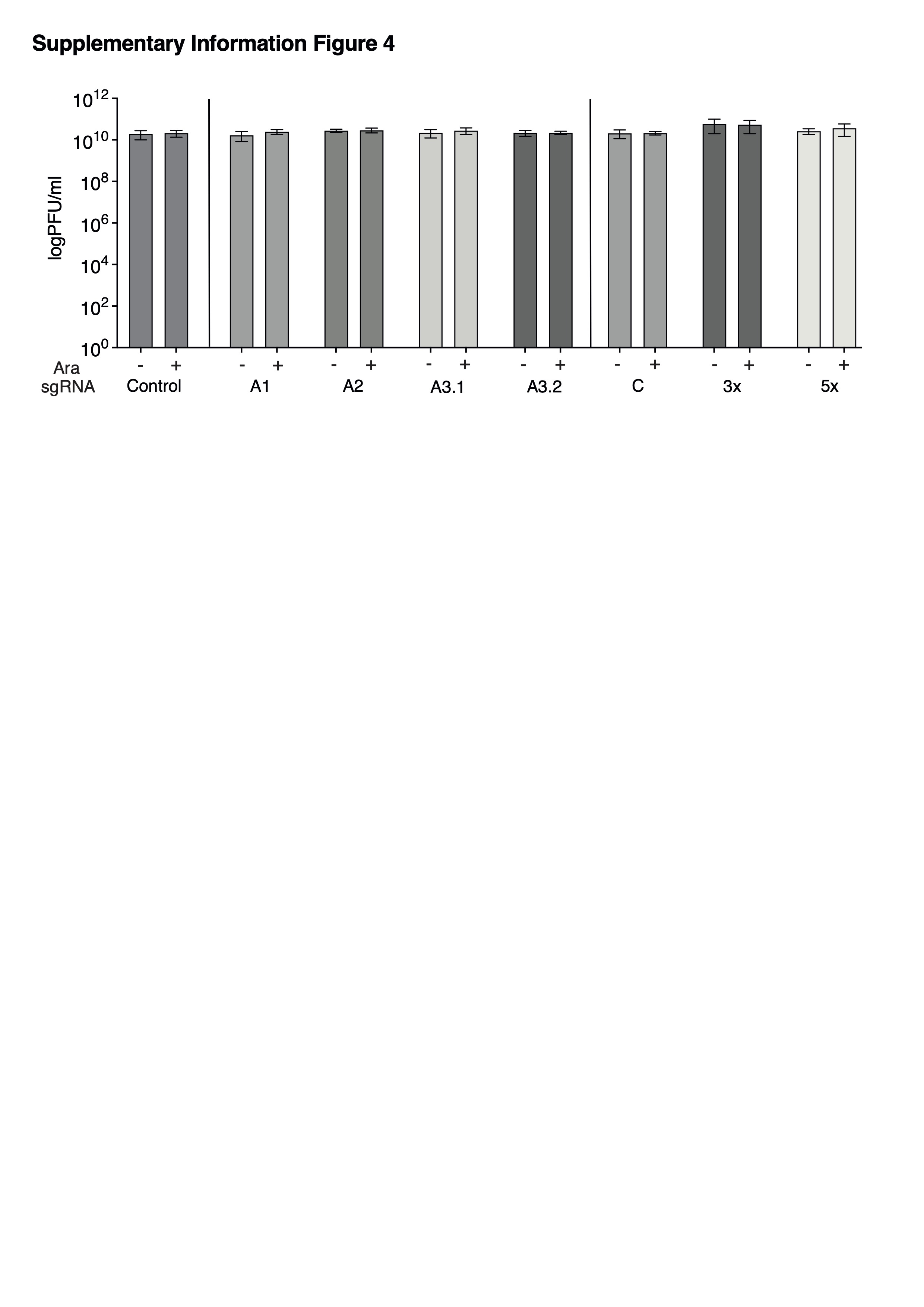

Supplement: SUPPLEMENTARY FIGURE 4 — Efficiency of plaquing (EOP) of phage T7 on strains expressing sgRNAs. Shown are EOPs of phage T7 growing on TB549 top agar in the absence or presence of the dCas9-inducer L-arabinose (0.1%). There is no statistical significance between uninduced and induced dCas9 expression on EOP (two-tailed t-tests). The means of three independent experiments are shown. Error bars are one standard error of the mean. [file Image_4.jpeg]

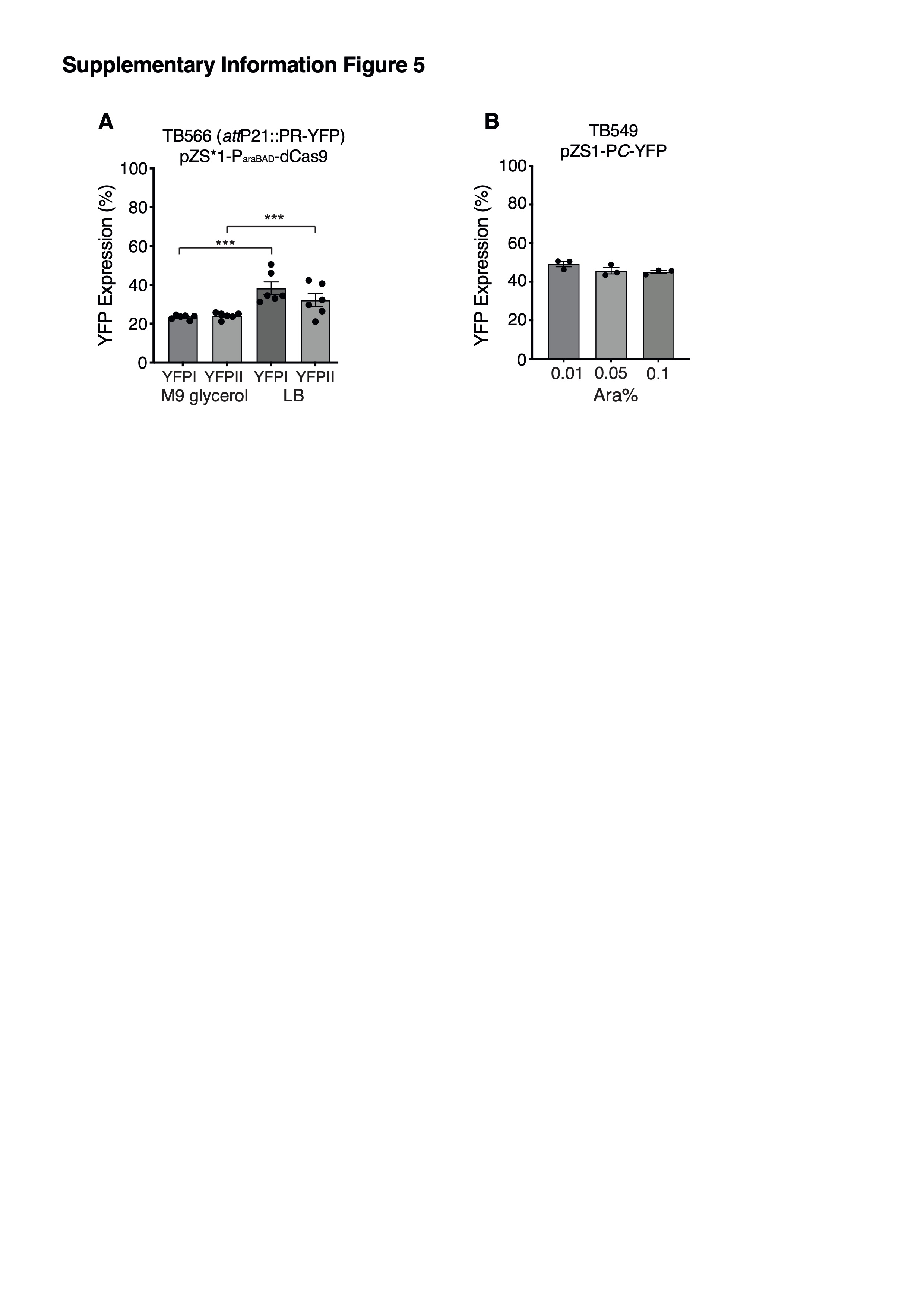

Supplement: SUPPLEMENTARY FIGURE 5 — CRISPRi knockdown efficacy of a single-copy target and at different dCas9 induction levels. (A) CRISPRi knockdown efficacy when targeting a single copy and constitutively expressed YFP. Shown is knockdown or PR-YFP inserted in attP21 in strain TB566 carrying a very low copy pZS*1-ParaBAD-dCas9 plasmid induced with 0.1% arabinose using two different sgRNAs, YFPI and YFPII. Apparent knockdown efficiency in M9 glycerol is near 80%, and about 65% in LB broth. T-tests between indicated pairs are highly significant (p < 0.005). (B) Knockdown efficacy of promoter C across 0.01, 0.05 and 0.1% arabinose show no significant changes in YFP fluorescence levels. [file Image_5.jpeg]
